# Lack of a bridge between screening and medical management for hypertension: health screening cohort in Japan

**DOI:** 10.1186/s12889-020-09532-5

**Published:** 2020-09-17

**Authors:** Shingo Fukuma, Tatsuyoshi Ikenoue, Yoshiyuki Saito, Yukari Yamada, Yusuke Saigusa, Toshihiro Misumi, Masataka Taguri

**Affiliations:** 1 Human Health Sciences, Kyoto University Graduate School of Medicine, 53 Shogoin-Kawahara, Sakyo, Kyoto, 606-8507 Japan; 2 Department of Biostatistics, Yokohama City University School of Medicine, Yokohama, Japan; 3 Department of Data Science, Yokohama City University School of Data Science, Yokohama, Japan

**Keywords:** Hypertension, Untreated, Blood pressure management

## Abstract

**Background:**

Patient journeys for hypertensive individuals after detection at screening have not been well examined in a general population. Thus, we aimed to assess the medical treatment status and subsequent longitudinal changes in blood pressure in a middle-aged Japanese population.

**Methods:**

We conducted a cohort study using a nationwide Japanese health screening cohort, from April 2014 to March 2019. Among health screening participants aged 40–74 years who had not previously received treatment for hypertension, hypertensive patients were newly identified based on screening results, and their medical treatment status for hypertension during the year following their initial screening was assessed. The main outcomes were longitudinal changes in systolic blood pressure (SBP) and diastolic blood pressure (DBP) over 4 years after initial screening.

**Results:**

Of the 153,523 screening participants (mean age = 49.7 years), 16,720 (10.9%) and 4150 (2.7%) were newly detected as having hypertension, with baseline SBP of 140–159 mmHg (grade 1) and ≥ 160 mmHg (grade 2–3), respectively. Among them, 15.9% of the grade 1 hypertensive participants and 36.3% of the grade 2–3 hypertensive participants started receiving medical treatment during the year following initial screening. A linear generalised estimating equation with propensity score matching showed that receiving medical treatment was associated with 5.77 mmHg lower SBP (95% CI − 6.64 to − 4.90) and 3.82 mmHg lower DBP (95% CI − 4.47 to − 3.16) in the grade 1 hypertensive group, and 14.69 mmHg lower SBP (95% CI − 16.35 to − 13.04) and 8.42 mmHg lower DBP (95% CI − 9.49 to − 7.34) in the grade 2–3 hypertensive group.

**Conclusions:**

Health screenings detected hypertension in a substantial percentage of the middle-aged population in this study. However, detection was often followed by insufficient medical treatment and inappropriate blood pressure management. These findings indicate an inadequate link between health screenings and medical treatments in patients with hypertension.

## Clinical perspective

### What is new?

- Using nationwide large-scale data combining health screening results and medical claims longitudinally, we assessed patient journeys for hypertensive participants after detection at screenings. Among middle-aged screening participants, 10.9 and 2.7% were detected to have grade 1 (SBP 140–159 mmHg) or grade 2–3 (SBP ≥160 mmHg) hypertension, respectively. Only 15.9% of grade 1 hypertensive and 36.3% of grade 2–3 hypertensive participants had started medical treatment after 1 year from initial screening.
- Medical treatment after screening was associated with better blood pressure management for the subsequent 4 years, compared with non-treatment.

### What are the clinical implications?

- A substantial percentage of hypertensive participants could be newly detected at health screenings. However, the detection of hypertension was often followed by insufficient medical treatment and inappropriate blood pressure management. To maximise the effectiveness of a screening programme and improve population health outcomes for hypertensive patients, screening programmes need to be redesigned to ensure continuity of care from screening to medical intervention.

## Background

The number of patients with hypertension, representing a major public health issue, is increasing globally. The global prevalence of hypertension is reported to be as high as 25% [1, 2]. Cases of undiagnosed or poorly treated hypertensive patients are commonly reported, as their symptoms are unidentified until the disease has progressed [1, 3–5]. Undiagnosed individuals lack the opportunity to receive appropriate treatment, and their disease management and long-term prognosis may deteriorate. A detailed analysis is necessary to determine the number of patients from the general population among whom these diseases can be detected during health screenings, what medical follow-up treatment they receive, and how they manage blood pressure.

Various efforts are being made globally concerning screening, diagnosis, and disease management of hypertension. In Japan, a screening programme for metabolic syndromes—including hypertension—has operated nationally since 2008 [6]. According to government reports, 29 million people (more than half of the eligible population) underwent screenings in Japan; however, whether these screenings are followed by appropriate medical treatment after detection has not been fully verified. Thus, whether this system contributes to the appropriate management of hypertension is unknown. There is insufficient evidence and disagreement concerning the benefits of universal health screenings, such as those performed in Japan.

This study analysed medical claims and health screening data of middle-aged adults from a nationwide health screening database in Japan, estimated the prevalence of hypertension newly detected at health screenings, and described patients’ medical treatment status after screenings. Furthermore, we analysed the associations between treatment status and blood pressure in patients identified during screenings as having hypertension.

## Methods

### Data source

Health screening and medical claims data were extracted from the Health Insurance Association for Architecture and Civil Engineering companies (HIA^2^CE), one of the largest employment-based health insurance associations in Japan. HIA^2^CE provides health insurance to approximately 400,000 insured individuals in approximately 1800 construction-related companies throughout Japan, from large general contractors to small regional construction companies.

In Japan, all adults 40 years or older must receive a general annual health screening for metabolic syndromes. We defined the initial health screening as the first screening since April 2014, to define baseline variables. We used the second and subsequent health screenings as follow-up screenings to define outcome variables. The end of the follow-up period was March 2019. Data included items such as body mass index (BMI), haemoglobin A1c (HbA1c), systolic blood pressure (SBP), diastolic blood pressure (DBP), low-density lipoprotein (LDL) cholesterol, current smoking status, exercise habits, and alcohol consumption.

In Japan, all residents are insured by one of the public health insurance plans, which cover virtually all care including medical care, dental care, long-term care, and some preventive care like health screening. The public health insurance plans are basically either an employment basis or a community basis, depending on the individual employment status. Much of the health care costs (70–90%) are covered by public funds, and the rest is either paid out of pocket or covered by private supplementary health insurance [7]. Under universal health insurance, patients are free to choose their healthcare provider without restrictions. This situation provides a unique opportunity to observe the status of medical treatment after the detection of disease under conditions with the lesser financial burden of health care costs when compared to other countries, such as the US. Due to Japan’s universal health insurance system, medical claims data include all medical records of insured persons. Medical claims data are recorded monthly and include diagnosis and treatment details. Medical treatment status data were extracted from medical claims records during the 6 months before the initial screening to define previous treatment status, and from medical claims during the year following the initial screening to define treatment status after screening.

### Participants

We conducted a cohort study in which health screening data and medical claims data were integrated longitudinally. The study participants had received health screenings and were aged between 40 and 74 years. There were 199,534 individuals in this age group who received initial health screenings; however, 46,011 were excluded due to a treatment history for hypertension. If previous medical claims reflected a medical treatment history with hypertension-related disease codes (International Classification of Diseases, 10th revision [ICD-10] codes), the patient was defined as being previously treated for hypertension. Supplement eTable 1 shows the ICD-10 disease code list for defining treated diseases. If a participant was no longer insured with HIA^2^CE after 1 year, we excluded him/her from the analysis.

### Defining hypertension at screening

Initial health screening data (the first health screening since April 2014) were used to define hypertension at health screenings. To ensure accuracy, the measurement procedures of blood pressure at health screenings are standardised according to the government’s guidelines [6, 8]. As a rule, blood pressure is basically measured twice, and the average value is adopted. An SBP 140–159 mmHg and SBP ≥160 mmHg indicated grade 1 and grade 2–3 hypertension, respectively [8, 9]. Of the 153,523 eligible participants, individuals identified as being grade 1 hypertensive (*n* = 16,720) or grade 2–3 hypertensive (*n* = 4150) at the screening were included.

### Defining non-treatment and outcomes

For patients diagnosed with hypertension at the initial screening, their medical treatment status after the initial screening was determined using medical claims data for 1 year following the initial screening. If the patient received medical treatment for any hypertension-related disease code during that year, that patient was defined as receiving medical treatment for hypertension after the screening. According to the definition, medical treatment includes both behavioural interventions and pharmaceutical therapies.

Our main outcomes were based on SBP and DBP (mmHg), which were obtained from the second to fourth annual health screenings (repeated measures of blood pressure levels).

### Statistical analysis

Using the initial health screening data, we estimated the prevalence of hypertension detection at the screenings. Participants’ characteristics based on SBP levels at screenings were considered. We described the cumulative probability of being treated for hypertension by baseline SBP levels, using the Kaplan-Meier method.

Following propensity score matching, we assessed the associations between treatment and blood pressure control [10]. We calculated the propensity score of the probability of treatment using a logistic model including age, gender, BMI, HbA1c, SBP, DBP, current smoking status, alcohol habits, exercise habits, and comorbidities (diabetes, cerebrovascular disease, peripheral vascular disease, chronic lung disease, cancer, liver disease, peptic ulcer, insomnia, and depression). We selected those variables that were clinically relevant to the outcome [11] of blood pressure. After obtaining a 1:1 nearest neighbour propensity score matching with replacement within the calliper distance of 0.10, we used linear generalised estimating equations with robust variance [12, 13], and estimated mean difference as the average treatment effect on the entire population (ATE). In the model, we assumed the common mean differences of blood pressure levels between groups over 4 time points (year1 to year 4). We reported the robust Abadie-Imbens standard errors using the Stata “teffects psmatch” program [14, 15]. During propensity score matching, the ATE indicates the average difference in predicted outcomes between treatment statuses among a matched population (or “common support”) [10, 16]. We calculated the standardised difference for all variables in the model to assess the covariate balance between groups after matching [17]. A balance plot for propensity scores to assess the covariate balance after propensity score matching was examined (eFig. 1).

We conducted complete case analysis in our main analysis. To assess the effect of missing data of covariates, we performed an analysis in which the covariates with 3% or greater missing values were excluded from the model, to estimate propensity scores. In another sensitivity analysis to assess the differences at the final follow-up results (year 4), we assessed associations between treatment statuses and blood pressure levels for years later using generalised linear models with propensity score matching. In another sensitivity analysis, to assess the robustness to the classification of hypertension, we conducted an analysis using the other classification based both on SBP and DBP (grade 1 hypertension: SBP 140–159 mmHg or DBP 90–99 mmHg, grade 2–3 hypertension: SBP ≥160 mmHg or DBP ≥100 mmHg). Finally, to discuss generalisability of the study participants, the prevalence of hypertension and antihypertensive drug use in the health screening cohort were compared with those in the Japanese general population.

To evaluate the status of blood pressure during follow-up, we also described proportions of patients with controlled blood pressure level (SBP < 140 mmHg and DBP < 90 mmHg) over 4 years by treatment status (eTable 5).

All analyses were performed using Stata version 15.1 (StataCorp, College Station, TX, USA). All tests were 2-tailed; a significance level of *p* < 0.05 was used.

## Results

### Participant characteristics

**Fig. 1:**
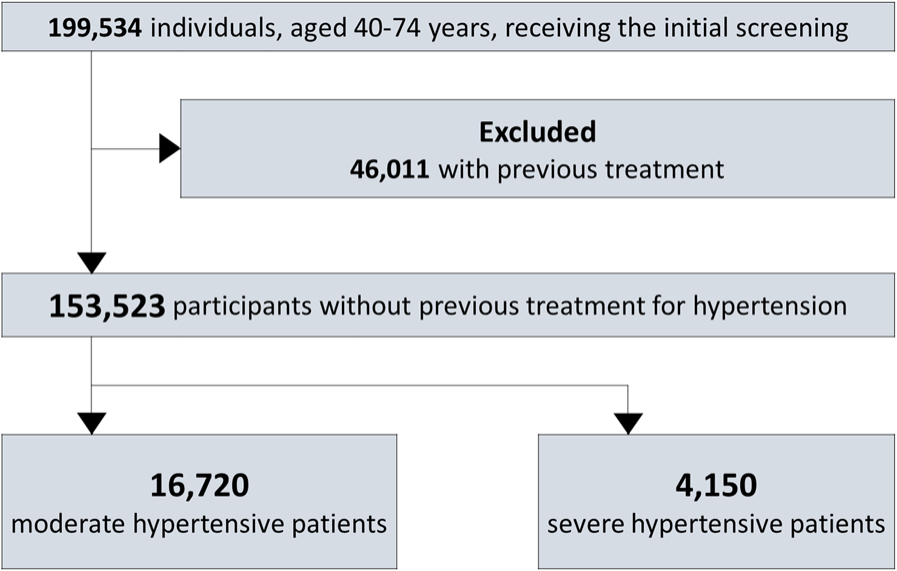
Selection process of study participants

Figure 1 shows a flow diagram of the participants. The final sample included 153,523 individuals with no previous treatment history of hypertension, who received initial health screenings. Participants’ mean age was 49.8 years, and 64.8% were male (Table 1). At the screening, we identified 16,720 (10.9%) grade 1 hypertensive participants (SBP 140–159 mmHg) and 4150 (2.7%) grade 2–3 hypertensive participants (SBP 160 mmHg or greater). Participants with higher SBP were generally older, had higher BMI, HbA1c, and LDL cholesterol, and were more likely to be male and smoke. The prevalence of hypertension and the percentage of antihypertensive drug use was lower in the cohort when compared to the general Japanese population (eTable 4).

**Table 1:** Baseline participant characteristics in total participants and subgroups stratified by baseline systolic blood pressure level

Variables	Total	Baseline systolic blood pressure		
	*N* = 153,523	< 140 mmHg *n* = 132,653	140–159 mmHg *n* = 16,720	≥160 mmHg *n* = 4150
Age, years	49.8 (8.3)	49.2 (8.1)	53.0 (8.8)	54.5 (8.6)
BMI, kg/m^2^	23.3 (3.6)	23.0 (3.5)	24.9 (3.9)	25.3 (4.3)
HbA1c, %	5.6 (0.6)	5.5 (0.6)	5.7 (0.8)	5.8 (1.1)
Systolic blood pressure, mmHg	122.6 (16.7)	118.0 (12.0)	147.1 (5.5)	171.3 (11.6)
Diastolic blood pressure, mmHg	75.9 (12.0)	73.3 (9.9)	90.4 (8.9)	101.2 (11.9)
LDL cholesterol, mg/dL	127.2 (32.2)	126.6 (31.9)	131.0 (33.9)	132.6 (35.4)
		*n* (%)	*n* (%)	*n (%)*
Men	99,474 (64.8)	83,287 (62.8)	12,972 (77.6)	3215 (77.5)
Current smoking	45,280 (29.5)	37,955 (28.6)	5744 (34.4)	1581 (38.1)
Drinking alcohol: Not everyday	89,877 (67.4)	79,862 (69.0)	8094 (57.5)	1921 (54.9)
Drinking alcohol: Everyday, small amount	30,537 (22.9)	25,752 (22.3)	3822 (27.1)	963 (27.5)
Drinking alcohol: Everyday, large amount	12,895 (9.7)	10,113 (8.7)	2167 (15.4)	615 (17.6)
Diagnosed comorbidities				
Diabetes	3015 (2.0)	2474 (1.9)	445 (2.7)	96 (2.3)
Cancer	2845 (1.9)	2493 (1.9)	295 (1.8)	57 (1.4)
Chronic lung disease	7879 (5.1)	7059 (5.3)	701 (4.2)	119 (2.9)
Liver disease	6092 (4.0)	5238 (4.0)	716 (4.3)	138 (3.3)
Peptic ulcer	6119 (4.0)	5356 (4.0)	654 (3.9)	109 (2.6)
Cerebrovascular disease	1677 (1.1)	1476 (1.1)	172 (1.0)	29 (0.7)
Peripheral vascular disease	1737 (1.1)	1521 (1.2)	181 (1.1)	35 (0.8)
Insomnia	5492 (3.6)	4974 (3.8)	455 (2.7)	63 (1.5)
Depression	3093 (2.0)	2851 (2.2)	217 (1.3)	25 (0.6)

*Abbreviations: M* mean, *SD* standard deviation, *BMI* body mass index

### Medical treatment after detection at screening

**Fig. 2:**
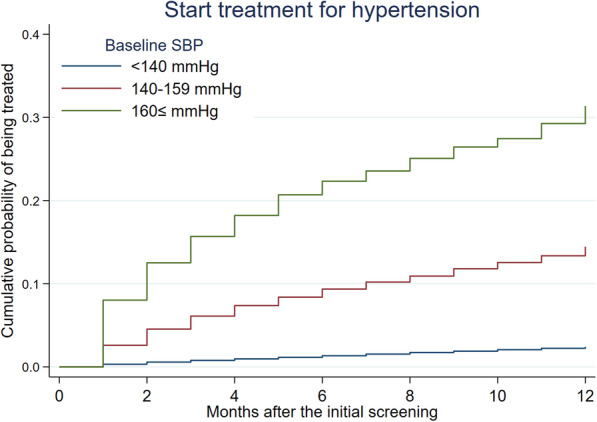
Cumulative probability of being treated for hypertension by baseline systolic blood pressure level

Among hypertensive participants with 209,751 person-month follow-ups, 3460 individuals started treatment for hypertension, and the incidence rate of starting treatment was 16.5 per 1000 person-years (95% confidence interval [CI] 16.0 to 17.1). Higher SBP at baseline was associated with higher incidence of starting treatment (Fig. 2). At 1 year after the initial screening, 15.9% (2666/16,720) and 36.3% (1506/4150) of participants had received treatment for grade 1 or grade 2–3 hypertension, respectively. Among those who started treatment, 53.6, and 68.1% received antihypertensive medications at the second screening in grade 1 and grade 2–3 hypertensive groups, respectively. Among hypertensive participants, the treatment group was generally older and had higher SBP and DBP (eTable 2).

### Longitudinal changes in blood pressure after screening by treatment status

**Fig. 3:**
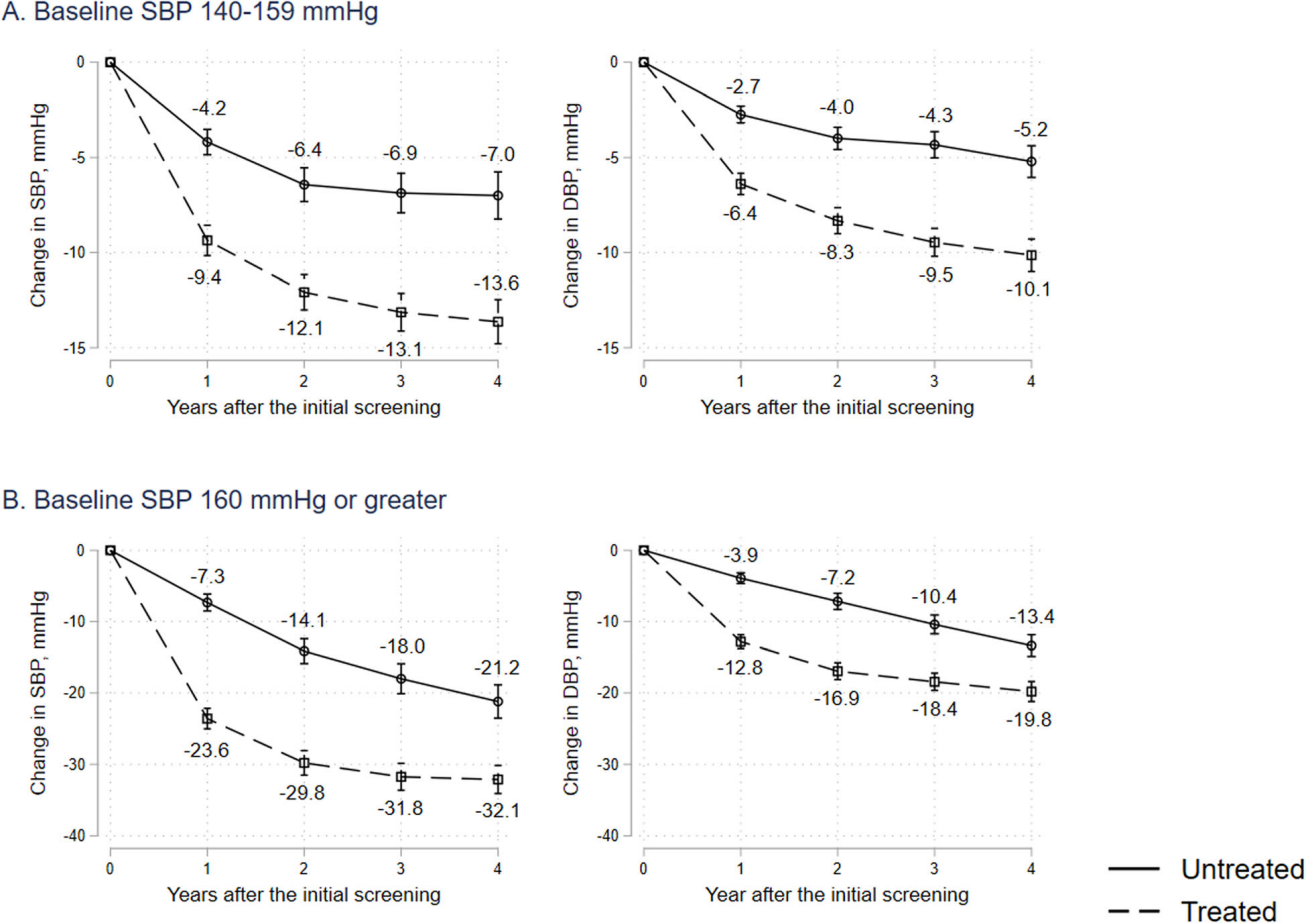
Longitudinal change in blood pressure after initial screening by treatment status among propensity matched cohort

Among hypertensive participants detected at the initial screening (year 0), we found longitudinal declines in SBP and DBP (year 1 to year 4) in both untreated and treated participants (Fig. 3). Differences in SBP and DBP between treated and untreated groups, as defined by treatment status from years 0 to 1, continued for 4 years. We found consistent differences both in SBP and DBP. The differences were greater in the grade 2–3 hypertensive group compared with the grade 1 hypertensive group.

### Associations between treatment status and blood pressure for 4 years

Using a 1:1 nearest neighbour propensity score matching within the calliper distance of 0.10, we obtained 1539 and 811 matched pairs in the grade 1 and grade 2–3 hypertensive group, respectively. As a result, we included 3078 (1539 pairs) and 1622 (811 pairs) participants in propensity score matching analysis in the grade 1 and grade 2–3 hypertensive group, respectively.

**Fig. 4:**
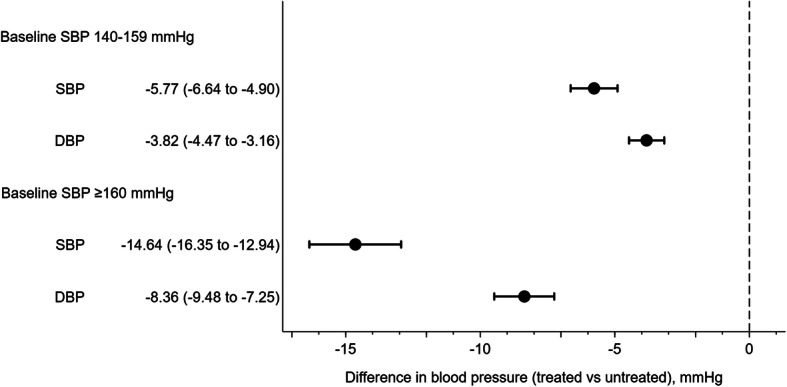
Treatment status and subsequent blood pressure levels stratified by baseline systolic blood pressure level. We estimated differences in systolic blood pressure (SBP) and diastolic blood pressure (DBP) for 4 years between treated and untreated groups among hypertensive participants, using linear generalized estimating equations with propensity score matching

The propensity score matching analysis of grade1 hypertensive participants (*n* = 3078) revealed that the treatment group had lower SBP (difference − 5.77 mmHg, 95% CI [− 6.64, − 4.90], *p* < 0.01) and lower DBP (difference − 3.82 mmHg, 95% CI [− 4.47, − 3.16], *p* < 0.01), compared with the non-treatment group. Among grade 2–3 hypertensive participants (*n* = 1622), the treatment group had lower SBP (difference − 14.64 mmHg, 95% CI [− 16.35, − 12.94], p < 0.01) and lower DBP (difference − 8.36 mmHg, 95% CI [− 9.48, − 7.25], p < 0.01), compared with the non-treatment group (Fig. 4).

Table 2 shows the balance of the participant characteristics after propensity score matching. The standardised differences for all covariates after matching ranged between − 0.1 and 0.1, indicating an improved covariate balance. eFigure1 shows that the distributions of propensity scores after matching were almost the same between two groups, indicating comparability after propensity score matching.

**Table 2:** Participant characteristics according to treatment status in hypertensive participants after propensity matching

Variables	Baseline systolic blood pressure 140–159 mmHg				Baseline systolic blood pressure ≥ 160 mmHg			
	Untreated *n* = 1539	Treated *n* = 1539	Standardized Difference^**a**^	***P*** value	Untreated *n* = 811	Treated *n* = 811	Standardized Difference^**a**^	***P*** Value
Age, years	53.3 (8.2)	53.4 (8.1)	−0.01	0.74	53.7 (8.5)	53.8 (8.2)	−0.01	0.84
BMI, kg/m^2^	25.4 (3.9)	25.3 (3.8)	0.01	0.85	25.7 (4.8)	25.7 (4.3)	−0.01	0.90
HbA1c, %	5.8 (1.0)	5.8 (1.0)	0.002	0.96	5.9 (1.0)	5.9 (1.2)	−0.06	0.22
Systolic blood pressure, mmHg	148.8 (5.6)	148.7 (5.6)	0.03	0.45	173.0 (12.0)	172.9 (12.1)	0.002	0.97
Diastolic blood pressure, mmHg	94.0 (8.4)	94.0 (9.4)	−0.003	0.94	104.3 (11.3)	104.2 (11.6)	0.02	0.76
LDL cholesterol, mg/dL	132.7 (33.8)	133.5 (34.3)	−0.02	0.53	133.1 (34.2)	133.0 (35.0)	0.001	0.99
	*n* (%)	*n* (%)			*n* (%)	*n* (%)		
Men	1269 (82.5)	1267 (82.3)	0.003	0.96	678 (83.6)	681 (84.0)	0.01	0.89
Current smoking	510 (33.1)	514 (33.4)	0.01	0.91	333 (41.1)	325 (40.1)	0.02	0.72
Drinking alcohol: Not everyday	835 (54.3)	845 (54.9)	0.01	0.94	428 (52.8)	423 (52.2)	0.04	0.74
Drinking alcohol: Everyday, small amount	452 (29.4)	444 (28.8)			233 (28.7)	246 (30.3)		
Drinking alcohol: Everyday, large amount	252 (16.4)	250 (16.2)			150 (18.5)	142 (17.5)		
Exercise habit	615 (40.0)	642 (41.7)	0.04	0.34	290 (35.8)	318 (39.2)	0.07	0.17
Diagnosed comorbidities								
Diabetes	41 (2.7)	33 (2.1)	0.03	0.41	18 (2.2)	22 (2.7)	0.03	0.63
Cancer	24 (1.6)	23 (1.5)	0.01	1.00	11 (1.4)	12 (1.5)	0.01	1.00
Chronic lung disease	70 (4.5)	66 (4.3)	0.01	0.79	31 (3.8)	24 (3.0)	0.05	0.41
Liver disease	86 (5.6)	72 (4.7)	0.04	0.29	29 (3.6)	23 (2.8)	0.04	0.48
Peptic ulcer	68 (4.4)	64 (4.2)	0.01	0.79	37 (4.6)	23 (2.8)	0.09	0.09
Cerebrovascular disease	15 (1.0)	17 (1.1)	0.01	0.86	8 (1.0)	5 (0.6)	0.04	0.58
Peripheral vascular disease	18 (1.2)	20 (1.3)	0.01	0.87	7 (0.9)	9 (1.1)	0.02	0.80
Insomnia	60 (3.9)	43 (2.8)	0.06	0.11	15 (1.8)	12 (1.5)	0.03	0.70
Depression	28 (1.8)	22 (1.4)	0.03	0.48	15 (1.8)	8 (1.0)	0.07	0.21

*Abbreviations: M* mean, *SD* standard deviation, *BMI* body mass index, SBP systolic blood pressure, DBP diastolic blood pressure

^a^reference category is the treatment group

### Sensitivity analysis

Even after excluding covariates with 3% or greater missing values (HbA1c 9.1%, alcohol habits 13.2%, exercise habits 15.3%), we found consistent associations between treatment status and blood pressure (eFigure 2). We found consistent differences in blood pressure levels between groups at the final follow-up results (year 4) (eFigure 3). Our findings were qualitatively unaffected by the use of the different classification of hypertension (eTable 3).

The proportions of participants with controlled blood pressure level were consistently higher in the treatment group than the non-treatment group over 4 years in both grade 1 and grade 2–3 hypertensive patients (eTable 5).

## Discussion

Among 153,523 middle-aged individuals without previous treatment for hypertension, 10.9 and 2.7% were detected as having grade 1 (SBP 140–159 mmHg) or grade 2–3 (160 mmHg or greater) hypertension, respectively. At 1 year after detection at screening, only 15.9 and 36.3% had started receiving treatment for hypertension among grade 1 or grade 2–3 hypertensive participants, respectively. Treatment after screening was associated with subsequent better blood pressure (SBP and DBP) management for 4 years. The results of this study pose important questions concerning the lack of sufficient medical management for hypertension after detections at health screenings.

### Lack of effective medical intervention after screening

This study analysed patients over time from screening and detection to medical treatment using real-world data. To our knowledge, this is the first large-scale longitudinal analysis of patients’ journeys after detections of hypertension at health screenings in a general population. Since medical claims and health screening data were longitudinally merged on an individual basis, we could define medical treatment status before and after screening, as well as follow-up health screening results, in clear chronological order. In this study, universal health screenings of middle-aged individuals demonstrated that many potentially hypertensive patients could be detected early [4, 18]. However, many individuals, for whom this disease was detected during screening, did not receive medical treatment. This seems to point to a lack of an effective post-screening intervention system. In Japan, a country with universal health insurance and free access to medical care, [7] receiving medical treatment is relatively easy. However, the medical care system alone may not be able to offer sufficient medical treatment. A new approach that links screening and detection to medical treatment is necessary. Otherwise, the effectiveness of screenings will remain limited.

Our results of low percentage of medical treatment are consistent with previous studies [19–21]. The grade 2–3 hypertensive group started medical treatment earlier than the grade1 hypertensive group, though both groups had lower percentage. The clinical guidelines recommend antihypertensive medications more strongly for severer hypertension, which might motivate the grade 2–3 hypertensive group to receive medical treatment. Further, the reasons for non-treatment may be compound. Notifying screening results and encouraging physician visits after the screening might be weak interventions. Patient factors may include lack of awareness of the risk of hypertension. Socioeconomic factors (such as income, family, and educational history) may influence behaviour of visiting physicians, [22] but such factors were not captured in the current study. Further, since the Japanese clinical guidelines for grade 1 hypertension recommend starting with lifestyle changes before receiving medications in grade 1 hypertensive patients, [8, 9] anti-hypertensive drug use in grade 1 hypertension may be less than in other countries [19]. The estimated effect in this study included both effects of non-pharmacological (such as lifestyle guidance) and pharmacological treatment. The magnitude of the estimated effects were consistent with previous studies [20, 21]. Future studies should examine the details of the treatment to gain a fuller picture.

### Clinical and public health implications

Our results indicated the clinical importance of medical treatment after detection of hypertension, as medical treatment was associated with substantial decreases in blood pressure over 4 years. Those population-level differences in blood pressure have a substantial impact on clinical outcomes for cardiovascular disease, cerebrovascular disease, and kidney disease [2, 22]. Medical treatment after screenings, such as drug administration and lifestyle improvement, may be effective, and a recently published study in China demonstrated lowering blood pressure through screenings [23]. From the perspective of public health, a high percentage of untreated patients after screenings indicates inefficiency in the screening programme. Efforts to strengthen the linkage between prevention and primary care are being considered in many countries, [8, 9, 24, 25] but have not yet been established. A new intervention design should be considered for patients detected at screenings to ensure they visit physicians appropriately [26].

### Limitations

This study has several limitations. First, while this observational study examined the associations between treatment status and subsequent blood pressure, it did not make random assignments for treatment status. Therefore, differences in participant characteristics between the non-treatment and treatment groups might exist, which could confound the associations. However, our propensity score analysis improved the balance parameters after matching. We also found that the balance plot of propensity scores improved after matching. Second, the database used in this study included more men than women. We only analysed participants who received the health screening, and the information about non-participants was unavailable. Screened participants may be healthier than non-participants. Given that our study focused on screened participants in Japanese employment-based health insurance (many of them are working-age men), our findings may not be generalisable to non-employees or to populations of other countries. Third, this study used annual health screening data to identify participants and define their blood pressure results. Participants’ blood pressure measured during screening may have fluctuations between measurements and might be affected by measurement conditions. Fourth, we were not able to assess details of medical treatments, as we extracted diagnosis codes, but not procedure codes or medication codes, from medical claims. Therefore, we could not discuss variations in medical treatments, such as type, dose, and number of anti-hypertensive drugs. Further, information on compliance to the drugs were not available from medical claims data. Given that some patients might not take the prescribed drugs, the estimated effect of treatments could be under-estimated. Fifth, participants with higher cardiovascular risk may be more likely to visit physicians seeking antihypertensive medications, compared with those with lower cardiovascular risk. The residual confounding due to unmeasured variable of cardiovascular risk may lead to an overestimation of the treatment effect, even though we used propensity score matching. There may be some unmeasured confounding variables, such as geographical and socioeconomic variables. Future research to assess such variations may be needed. Finally, we were not able to assess other interventions outside health insurance, which may affect change in blood pressure. Some patients might receive traditional medicines and join health promotion activities. Those treatments and activities were not recorded in the medical claims database.

## Conclusions

Many middle-aged individuals with hypertension can be detected by health screenings; however, these screenings are often followed by insufficient medical treatment and poor blood pressure management. These findings indicated the lack of a bridge between health screenings and medical treatments in patients with hypertension. To maximise the effectiveness of screening programmes and improve population health for hypertensive patients, screening programmes need to be redesigned to ensure continuity of care from screening to medical intervention.

## Supplementary information

**Additional file 1: eTable 1**. ICD-10 Codes that defined diseases in the medical claims data. **eTable 2**. Participant characteristics according to treatment status in hypertensive participants before propensity matching (total participants). **eFigure 1**. Balance plots of propensity scores before and after matching. The density plots on the left (Raw: before matching) indicate balance of propensity scores between treatment and non-treatment in total participants, and the density plots on the right (Matched: after matching) indicate balance of propensity scores between treatment status in propensity scores for matched participants**. eFigure 2**. Sensitivity analysis excluding covariates with 3% or greater missing values (HbA1c 9.1%, alcohol habit 13.2%, exercise habit 15.3%). We estimated differences in systolic blood pressure (SBP) and diastolic blood pressure (DBP) for 4 years between treated and untreated groups among hypertensive participants, using linear generalized estimating equations with propensity score matching. For propensity score matched participants, we included 3860 (1930 treated and 1930 untreated) and 2046 (1023 treated and 1023 untreated) individuals from the subgroup with baseline SBP 140–159 mmHg and subgroup with baseline SBP ≥160 mmHg, respectively. **eFigure 3**. Sensitivity analysis to assess the differences at the final follow-up results (year 4). We estimated differences in systolic blood pressure (SBP) and diastolic blood pressure (DBP) 4 years later between treated and untreated groups among hypertensive participants, using generalized linear models with propensity score matching. **eTable 3**. Sensitivity analysis using the other classification based both on systolic blood pressure (SBP) and diastolic blood pressure (DBP) (grade 1 hypertension: SBP 140–159 mmHg or DBP 90–99 mmHg, grade 2–3 hypertension: SBP ≥160 mmHg or DBP ≥100 mmHg). We estimated differences in systolic blood pressure (SBP) and diastolic blood pressure (DBP) between treated and untreated groups among hypertensive participants, using generalized linear models with propensity score matching. **eTable 4**. To assess generalizability of the study participants, the prevalence of hypertension and antihypertensive drug use in the health screening cohort were compared with those in the Japanese general population**. eTable 5**. Proportion of patients with blood pressure level < 140/90 mmHg during follow-up.

## Data Availability

The datasets generated and/or analysed during the current study are not publicly available due to restrictions from the Health Insurance Association for Architecture and Civil Engineering companies.

## Abbreviations

SBP: systolic blood pressure
DBP: diastolic blood pressure
BMI: body mass index
HbA1c: haemoglobin A1c
LDL: low-density lipoprotein
