# Supplementary material for: Lack of a bridge between screening and medical management for hypertension: health screening cohort in Japan
Source: BMC Public Health. 2020 Sep 17;20:1419. doi: 10.1186/s12889-020-09532-5 (PMC7499996; doi:10.1186/s12889-020-09532-5)
Supplement: Supplementary file 1 — Additional file 1: eTable 1. ICD-10 Codes that defined diseases in the medical claims data. eTable 2. Participant characteristics according to treatment status in hypertensive participants before propensity matching (total participants). eFigure 1. Balance plots of propensity scores before and after matching. The density plots on the left (Raw: before matching) indicate balance of propensity scores between treatment and non-treatment in total participants, and the density plots on the right (Matched: after matching) indicate balance of propensity scores between treatment status in propensity scores for matched participants. eFigure 2. Sensitivity analysis excluding covariates with 3% or greater missing values (HbA1c 9.1%, alcohol habit 13.2%, exercise habit 15.3%). We estimated differences in systolic blood pressure (SBP) and diastolic blood pressure (DBP) for 4 years between treated and untreated groups among hypertensive participants, using linear generalized estimating equations with propensity score matching. For propensity score matched participants, we included 3860 (1930 treated and 1930 untreated) and 2046 (1023 treated and 1023 untreated) individuals from the subgroup with baseline SBP 140–159 mmHg and subgroup with baseline SBP ≥160 mmHg, respectively. eFigure 3. Sensitivity analysis to assess the differences at the final follow-up results (year 4). We estimated differences in systolic blood pressure (SBP) and diastolic blood pressure (DBP) 4 years later between treated and untreated groups among hypertensive participants, using generalized linear models with propensity score matching. eTable 3. Sensitivity analysis using the other classification based both on systolic blood pressure (SBP) and diastolic blood pressure (DBP) (grade 1 hypertension: SBP 140–159 mmHg or DBP 90–99 mmHg, grade 2–3 hypertension: SBP ≥160 mmHg or DBP ≥100 mmHg). We estimated differences in systolic blood pressure (SBP) and diastolic blood pressure (DBP) between treated and untreat [file 12889_2020_9532_MOESM1_ESM.docx]

**Supplement**

**Lack of a bridge between screening and medical management for hypertension:**

**health screening cohort in Japan**

Shingo Fukuma^1^, Tatsuyoshi Ikenoue^1^, Yoshiyuki Saito^1^, Yukari Yamada ^1^, Yusuke Saigusa^2^,

Toshihiro Misumi^2^, and Masataka Taguri^3^

^1^Human Health Sciences, Kyoto University Graduate School of Medicine, Kyoto, Japan; ^2^Department of Biostatistics, Yokohama City University School of Medicine, Yokohama, Japan; ^3^Department of Data Science, Yokohama City University School of Data Science, Yokohama, Japan

**Corresponding Author:**

Shingo Fukuma, MD, PhD

Human Health Sciences, Kyoto University Graduate School of Medicine

53 Shogoin-Kawahara, Sakyo, Kyoto 606-8507, Japan

E-mail: fukuma.shingo.3m@kyoto-u.ac.jp

Phone: +81-75-751-3925

Fax: +81-75-751-3909

**eTable 1.** ICD-10 Codes that defined diseases in the medical claims data

| **Disease** | **ICD-10 codes** |
| --- | --- |
| Hypertension | D35.0, E87.5, I10, I11.0, I11.9, I12.0, I12.9, I13.9, I15.0, I15.1, I15.2, I15.8, I15.9, I61.9 |
| Diabetes | E10, E10.0, E10.1, E10.2, E10.3, E10.4, E10.5, E10.6, E10.7, E10.9, E11, E11.0, E11.1, E11.2, E11.3, E11.4, E11.5, E11.6, E11.7, E11.9, E12, E13, E13.0, E13.1, E13.2, E13.3, E13.4, E13.5, E13.6, E13.7, E13.9, E14, E14.0, E14.1, E14.2, E14.3, E14.4, E14.5, E14.6, E14.9, E88.1, E88.8 |
| Cancer | C00.x-C26.x, C30.x-C34.x, C37.x-C41x, C45.x-C58.x, C60.x-C76.x, C81.x-C85.x, C88.3, C88.7, C88.9, C90.0, C90.1, C91.x- C93.x, C94.0-C94.3, C94.5, C94.7, C95.x, C96.x, C43.x, C88.0-C88.2, C90.2, C94.4, C97.x |
| Chronic lung disease | J41.x-J47.x, J60.x-J66.x, I27.8, I27.9, J40.x, J67.x, J68.4, J70.1, J70.3 |
| Liver disease | K70.3, K71.7, K73.x, K74.3- K74.6, B18.x, K70.0-K70.2, K70.9, K71.3-K71.5, K74.0-K74.2, K76.0, K76.2-K76.4, K76.8, K76.9, Z94.4, K72.1, K72.9, K76.6, K76.7, I85.0, I85.9, I86.4, I98.2, K70.4, K71.1, K76.5 |
| Peptic ulcer | K25.4-K25.7, K26.4-K26.7, K27.4-K27.7, K28.4-K28.7, K25.0-K25.3, K25.9, K26.0-K26.3, K26.9, K27.0-K27.3, K27.9, K28.0-K28.3, K28.9 |
| Cerebrovascular disease | A18.8, A32.8, A52.0, E85.9, F01.1, G40.9, G45.0, G45.1, G45.3, G45.4, G45.8, G45.9, G81.9, H34.0, I60.0, I60.1, I60.2, I60.3, I60.4, I60.5, I60.6, I60.7, I60.8, I60.9, I61.0, I61.1, I61.3, I61.4, I61.5, I61.6, I61.8, I61.9, I62.0, I62.1, I62.9, I63.0, I63.1, I63.2, I63.3, I63.4, I63.5, I63.6, I63.8, I63.9, I64, I65.0, I65.1, I65.2, I65.3, I66.0, I66.1, I66.2, I66.3, I66.8, I66.9, I67.0, I67.1, I67.2, I67.3, I67.4, I67.5, I67.6, I67.7, I67.8, I67.9, I69.0, I69.1, I69.3, I69.4, O99.4 |
| Peripheral vascular disease | A52.0, E10.5, E11.5, E14.5, I70.0, I70.1, I70.2, I70.20, I70.21, I70.8, I70.9, I71.0, I71.1, I71.2, I71.3, I71.4, I71.5, I71.6, I71.8, I71.9, I73.1, I73.8, I73.9, I77.1, K55.1, K55.8, K55.9, T82.8, Z95.8 |
| Insomnia | G47.0, G47.2, G47.4, G47.8, G47.9 |
| Depression | F31.3, F31.4, F31.5, F31.6, F31.7, F31.9, F32.0, F32.1, F32.2, F32.3, F32.8, F32.9 |

Abbreviations: ICD-10, International Classification of Diseases, 10th revision

**eTable 2.** Participant characteristics according to treatment status in hypertensive participants before propensity matching (total participants)

| **Variables** | **Baseline systolic blood pressure**  **140-159 mmHg** | | | **Baseline systolic blood pressure**  **≥160 mmHg** | | |
| --- | --- | --- | --- | --- | --- | --- |
|  | **Untreated**  *n* = 14054 | **Treated**  *n* = 2666 | **Standardized**  **Difference^*^** | **Untreated**  *n* = 2644 | **Treated**  *n* = 1506 | **Standardized**  **Difference^*^** |
| Age, years | 52.8 (8.8) | 54.2 (8.5) | -0.16 | 54.4 (8.7) | 54.7 (8.5) | -0.03 |
| BMI, kg/m^2^ | 24.9 (3.9) | 25.2 (4.0) | -0.08 | 25.1 (4.3) | 25.6 (4.4) | -0.11 |
| HbA1c, % | 5.7 (0.8) | 5.8 (1.0) | -0.16 | 5.8 (0.9) | 5.9 (1.2) | -0.13 |
| Systolic blood pressure, mmHg | 146.8 (5.4) | 148.6 (5.6) | -0.32 | 170.2 (10.5) | 173.3 (13.1) | -0.26 |
| Diastolic blood pressure, mmHg | 89.9 (8.8) | 92.9 (9.4) | -0.33 | 99.9 (11.4) | 103.5 (12.3) | -0.30 |
| LDL cholesterol, mg/dL | 130.8 (33.7) | 132.3 (35.1) | -0.04 | 132.3 (35.6) | 133.2 (35.3) | -0.02 |
|  | *n* (%) | *n* (%) |  | *n* (%) | *n* (%) |  |
| Men | 10904 (77.6) | 2068 (77.6) | 0.0004 | 2057 (77.8) | 1158 (76.9) | 0.02 |
| Current smoking | 4856 (34.6) | 888 (33.3) | 0.03 | 998 (37.8) | 583 (38.7) | 0.02 |
| Drinking alcohol: Not everyday | 6801 (57.7) | 1293 (56.5) | 0.04 | 1225 (55.3) | 696 (54.2) | 0.07 |
| Drinking alcohol: Everyday, small amount | 3170 (26.9) | 652 (28.5) |  | 585 (26.4) | 378 (29.4) |  |
| Drinking alcohol: Everyday, large amount | 1824 (15.5) | 343 (15.0) |  | 404 (18.2) | 211 (16.4) |  |
| Exercise habit | 5043 (44.1) | 958 (43.2) | 0.02 | 936 (43.9) | 504 (40.4) | 0.07 |
| Diagnosed comorbidities |  |  |  |  |  |  |
| Diabetes | 390 (2.8) | 55 (2.1) | 0.03 | 66 (2.5) | 30 (2.0) | 0.03 |
| Cancer | 256 (1.8) | 39 (1.5) | 0.03 | 38 (1.4) | 19 (1.3) | 0.02 |
| Chronic lung disease | 596 (4.2) | 105 (3.9) | 0.03 | 72 (2.7) | 47 (3.1) | 0.02 |
| Liver disease | 601 (4.3) | 115 (4.3) | 0.002 | 91 (3.4) | 47 (3.1) | 0.02 |
| Peptic ulcer | 550 (3.9) | 104 (3.9) | 0.001 | 64 (2.4) | 45 (3.0) | 0.03 |
| Cerebrovascular disease | 140 (1.0) | 32 (1.2) | 0.02 | 21 (0.8) | 8 (0.5) | 0.03 |
| Peripheral vascular disease | 151 (1.1) | 30 (1.1) | 0.005 | 21 (0.8) | 14 (0.9) | 0.01 |
| Insomnia | 372 (2.6) | 83 (3.1) | 0.03 | 39 (1.5) | 24 (1.6) | 0.01 |
| Depression | 182 (1.3) | 35 (1.3) | 0.02 | 14 (0.5) | 11 (0.7) | 0.03 |

Abbreviations: *M,* mean; *SD*, standard deviation; BMI, body mass index

^*^reference category is the treated group

**eFigure 1.** Balance plots of propensity scores before and after matching. The density plots on the left (Raw: before matching) indicate balance of propensity scores between treatment and non-treatment in total participants, and the density plots on the right (Matched: after matching) indicate balance of propensity scores between treatment status in propensity scores for matched participants.

1. Baseline SBP 140-159 mmHg

1. Baseline SBP 160 mmHg or greater

**eFigure 2.** Sensitivity analysis excluding covariates with 3% or greater missing values (HbA1c 9.1%, alcohol habit 13.2%, exercise habit 15.3%). We estimated differences in systolic blood pressure (SBP) and diastolic blood pressure (DBP) for four years between treated and untreated groups among hypertensive participants, using linear generalized estimating equations with propensity score matching. For propensity score matched participants, we included 3860 (1930 treated and 1930 untreated) and 2046 (1023 treated and 1023 untreated) individuals from the subgroup with baseline SBP 140-159 mmHg and subgroup with baseline SBP ≥160 mmHg, respectively.

**eFigure 3.** Sensitivity analysis to assess the differences at the final follow-up results (year 4). We estimated differences in systolic blood pressure (SBP) and diastolic blood pressure (DBP) four years later between treated and untreated groups among hypertensive participants, using generalized linear models with propensity score matching.

**eTable 3.** Sensitivity analysis using the other classification based both on systolic blood pressure (SBP) and diastolic blood pressure (DBP) (grade 1 hypertension: SBP 140-159 mmHg or DBP 90-99 mmHg, grade 2-3 hypertension: SBP ≥160 mmHg or DBP ≥100 mmHg). We estimated differences in systolic blood pressure (SBP) and diastolic blood pressure (DBP) between treated and untreated groups among hypertensive participants, using generalized linear models with propensity score matching.

|  | **Grade 1 hypertension**  SBP 140-159 mmHg and/or  DBP 90-99 mmHg | **Grade 2-3 hypertension**  SBP ≥160 mmHg and/or  DBP ≥100 mmHg |
| --- | --- | --- |
| SBP, mmHg | -4.51  (-5.40 to -3.62) | -12.62  (-13.90 to -11.35) |
| DBP, mmHg | -2.75  (-3.37 to -2.13) | -7.85  (-8.65 to -7.05) |

**eTable 4.** To assess generalizability of the study participants, the prevalence of hypertension and antihypertensive drug use in the health screening cohort were compared with those in the Japanese general population.

|  | Health screening cohort  N=199,534 | | Japanese general population,  aged 40-74 years |
| --- | --- | --- | --- |
|  | Crude | Age-sex standardised^b^ |  |
| Hypertension^a^, % | 31.8% | 35.9% | 49.9% |
| Anti-hypertensive drug use, % | 16.9% | 21.3% | 25.8% |

^a^Hypertension was defined as SBP ≥140 mmHg, DBP ≥90 mmHg, or antihypertensive drug use.

^b^Adjusted for the 2017 age-gender distribution of the Japanese population aged 40–74 years. We extracted data from the online portal website for the official statistics of Japan (http://www.e-stat.go.jp/).

**eTable 5.** Proportion of patients with blood pressure level <140/90 mmHg during follow-up.

|  | **Grade 1 hypertension**  SBP 140-159 mmHg and/or  DBP 90-99 mmHg | | **Grade 2-3 hypertension**  SBP ≥160 mmHg and/or  DBP ≥100 mmHg | |
| --- | --- | --- | --- | --- |
|  | Untreated | Treated | Untreated | Treated |
| 1 year later | 33.8% | 42.3% | 8.2% | 28.8% |
| 2 years later | 36.0% | 50.9% | 13.6% | 37.6% |
| 3 years later | 38.3% | 54.1% | 20.7% | 44.2% |
| 4 years later | 39.8% | 54.9% | 27.4% | 47.4% |
